# Evaluation of four methods to identify the homozygotic sex chromosome in small populations

**DOI:** 10.1186/s12864-022-08393-z

**Published:** 2022-02-24

**Authors:** Charles Christian Riis Hansen, Kristen M. Westfall, Snæbjörn Pálsson

**Affiliations:** 1 Department of Life and Environmental Sciences, University of Iceland, Reykjavik, Iceland; 2 Current: Fisheries and Oceans Canada, Pacific Biological Station, Nanaimo, BC Canada

**Keywords:** Homogametic sex chromosome, Population genetics, Non-model organisms, White-tailed eagle

## Abstract

**Background:**

Whole genomes are commonly assembled into a collection of scaffolds and often lack annotations of autosomes, sex chromosomes, and organelle genomes (i.e., mitochondrial and chloroplast). As these chromosome types differ in effective population size and can have highly disparate evolutionary histories, it is imperative to take this information into account when analysing genomic variation. Here we assessed the accuracy of four methods for identifying the homogametic sex chromosome in a small population using two whole genome sequences (WGS) and 133 RAD sequences of white-tailed eagles (*Haliaeetus albicilla*): i) difference in read depth per scaffold in a male and a female, ii) heterozygosity per scaffold in a male and a female, iii) mapping to the reference genome of a related species (chicken) with annotated sex chromosomes, and iv) analysis of SNP-loadings from a principal components analysis (PCA), based on the low-depth RADseq data.

**Results:**

The best performing approach was the reference mapping (method iii), which identified 98.12% of the expected homogametic sex chromosome (Z). Read depth per scaffold (method i) identified 86.41% of the homogametic sex chromosome with few false positives. SNP-loading scores (method iv) identified 78.6% of the Z-chromosome and had a false positive discovery rate of more than 10%. Heterozygosity per scaffold (method ii) did not provide clear results due to a lack of diversity in both the Z and autosomal chromosomes, and potential interference from the heterogametic sex chromosome (W). The evaluation of these methods also revealed 10 Mb of putative PAR and gametologous regions.

**Conclusion:**

Identification of the homogametic sex chromosome in a small population is best accomplished by reference mapping or examining differences in read depth between sexes.

**Supplementary Information:**

The online version contains supplementary material available at 10.1186/s12864-022-08393-z.

## Background

Inferences about genetic variation, effective population size and population structure from genomic data in species that have heteromorphic sex chromosomes are dependent on their correct identification and other markers from different genomic regions, i.e., autosomes and the plastid genomes. As these different genomic regions typically have different ploidy numbers, substitution rates, and recombination rates, it follows that they will also be variably affected by genetic drift and selection [1]. Such information can be imperative for successful conservation management based on genetic variation and evolutionary studies. Annotating genomic regions can be accomplished either from a high-quality reference genome of the same or a closely related species. Here, we use genomic data from the white-tailed eagle mapped to a golden eagle reference genome to determine which scaffolds belong to the Z and autosomal chromosomes.

Using a different reference genome from the study species is frequently done [2–5] when chromosomal information is lacking. However, there can be drawbacks as related species can differ e.g., in genome size, synteny and other chromosomal rearrangements, or even lack sex chromosomes altogether [6]. Mapping to a closely related genome could also lead to mis-identification of a sequence that is sex-linked in the reference but not in the focal species or missing the sex chromosome content present in the focal that is not in the reference. However, birds are characterized by evolutionary stable chromosomes with rather little variation in genome size compared to other groups [7, 8].

Like many non-model species, the white-tailed eagle (*Haliaeetus albicilla*) lacks a well annotated genome. The specimens studied here come from a small and geographically isolated population of white-tailed eagles in Iceland, which currently consists of 80 breeding pairs. The population is recovering slowly from a severe bottleneck in population size during the 19th–20th centuries, when the number of breeding pairs were about 20 for more than 50 years [9] and is thus expected to have little genetic variation. The golden eagle (*Aquila chrysaetos*) and the white-tailed eagle are large raptors with a wide distribution in the northern hemisphere [10, 11]. Currently there are four genome assemblies available for the golden eagle, consisting of 142 (size: 1233.7 Mb, N50: 46.9 Mb); 1142 (size: 1192.7 Mb, N50: 9.2 Mb); 35,366 (size: 1196.0 Mb, N50: 0.11 Mb); and 42,881 (size: 1548.4 Mb, N50: 1.7 Mb) scaffolds, where only the first has scaffolds assigned to chromosomes [12]. Only three fragmented genomes exist for the white-tailed eagle (consisting of 50,905 scaffolds with the size: 1133.5 Mb, and N50: 0.05 Mb; 35,313 scaffolds with the size: 1196.5 Mb and N50: 0.12 Mb; and 6418 scaffolds with the size: 1222.6 Mb and N50: 4.5 Mb), with no annotated chromosomes [8]. The mitochondrial genomes of both the white-tailed and golden eagle have been identified [12, 13]. The Z-chromosome has been identified in golden eagle (*88.2 Mb*) and it is large in comparison with Z chromosomes in other birds which have been identified (ranging from 37.9 to 195.3 Mb [14–16]) but similar in size to chicken (*Gallus gallus*, 82.5 Mb [17]). Resolving the chromosomal composition of the white-tailed eagle genome will facilitate research on the genetics and evolutionary history of the species and for other eagle species. Furthermore, assessing the accuracy of methods for identifying the homozygotic sex chromosome may facilitate annotation of genome assemblies in other species characterized by small population sizes. Here we evaluate the success of four methods to identify the Z-chromosome in the small population of white-tailed eagles in Iceland. Sequencing depth (1) and patterns of heterozygosity (2) were analysed in high-depth whole genome sequence data obtained from one male and one female. The golden eagle scaffold reference genome was mapped to a chicken genome (3), and genotypes from low-depth RAD-sequencing data from 133 white-tailed eagles with a principal component analysis (PCA) (4). Our hypothesis is that the use of heterozygosity will be least successful as it will be reduced in the small population.

A recent review describes various methods for identifying sex chromosomes [18]. When template DNA molecules from a genome are sequenced randomly, it is expected that equivalent chromosomal classes will have similar average sequencing depths, and thus the depth can be used to identify different parts of the genome. For example, mitochondrial DNA is expected to have relatively high read depth, due to greater per-cell copy number than the nuclear chromosomes (this also applies to repeated regions). In addition, the sex chromosome found in the homogametic sex (ZZ or XX) is expected to have double the sequencing depth obtained from the heterogametic sex (ZW or XY), in species with differentiated sex chromosomes, as in birds and mammals [19, 20], but not in species with little differentiation between sex chromosomes such as in several fish species [21, 22]. Thus, for example, identification of the Z (and X) chromosome through depth filtering has been successfully applied to flycatchers [23] and humans [24], and depth is also partly used in programmes for discovering the sex chromosomes [25–27].

Sex differences in heterozygosity can also be used to assess which scaffolds belong to the homogametic sex chromosome e.g., [28]. For any given set of individuals from the same population, the Z-chromosome is expected to have fewer heterozygous positions in females (ZW) than in males (ZZ), whereas autosomal scaffolds are expected to have a similar number of heterozygous positions in both sexes.

Several factors can limit the discriminatory power of depth and heterozygosity to identify Z scaffolds when comparing males and females. First, the difference between the sexes will be reduced for scaffolds containing pseudoautosomal (PAR) and gametologous regions (conserved but non-recombining homologous regions). A study on PAR-regions in birds have shown large variation in the size and divergence of W- and Z-chromosomes across species [29], furthermore Xu and Zhou [19] showed that the W-chromosome has retained its gene function in birds better than the Y-chromosome in mammals and that the proportion of gametologs can be high. Moreover, long runs of homozygosity affecting Z scaffolds in males and autosomal scaffolds in both sexes, due to inbreeding or small population size, can mask the expected pattern of sex differences in heterozygosity. This is expected to be a marked feature of the white-tailed eagles analysed in this study and have a negative impact on how useful the heterozygosity is in identifying the Z-chromosome.

Another approach is to map scaffolds from an incompletely assembled reference genome to a more fully annotated genome from a “closely” related species. Such mapping can be done with several available programs e.g., LASTZ [30], LAST [31] and YASS [32]. The accuracy of chromosomal locations of scaffolds obtained from this approach depends on the evolutionary distance between the two reference genomes, which can differ due to chromosomal translocations, transposed regions, and repetitive regions [33, 34], sometimes even in closely related species [35]. Thus, this method may be only applicable for taxa with relatively stable genomes such as mammals and birds, though some groups of birds have also recently been shown to have dynamic sex chromosomes [36].

In a PCA of genotypes from all scaffolds i.e., belonging to both autosomes and sex-chromosomes, it is possible that one or more principal components (PCs) split males and females, due to sex specific markers on the sex chromosomes, i.e., on W or to markers on Z given a double weight in females. It therefore follows that a PCA could be used to identify scaffolds belonging to sex chromosomes, or alternatively to any sex specific markers, much in the same way as for population or group differentiation. Methods based on sex specific markers have been developed [37, 38] to identify the W and have been commonly used in PCR to diagnose sexes [39]. We tested this by examining the loadings of SNPs from a PCA based on low-depth RAD-sequencing data from 133 white-tailed eagles (Fig. S1) - to assess if they contribute to separation along a specific principal axis [40] by sex.

We show that sex differences in sequencing depth and mapping to a more complete reference genome from a related species provide the most effective means to identify Z chromosome scaffolds in the white-tailed eagles. However, the approaches based on the PCA, and heterozygosity provide valuable additional information and shed light on some key challenges faced by researchers working with genomic data from species with partially assembled reference genomes.

## Results

To assess the accuracy of the four approaches used to identify the Z-chromosomal scaffolds (depth, Heterozygosity, mapping, and PCA), the reference “scaffold-assembled” golden eagle genome was mapped to a newly released “chromosome-assembled” golden eagle genome, to know the position in the genome of the scaffolds. This was used as a baseline (“truth”) when evaluating the methods (Fig. 5, Table 2, and Method section).

### Depth

**Fig. 1:**
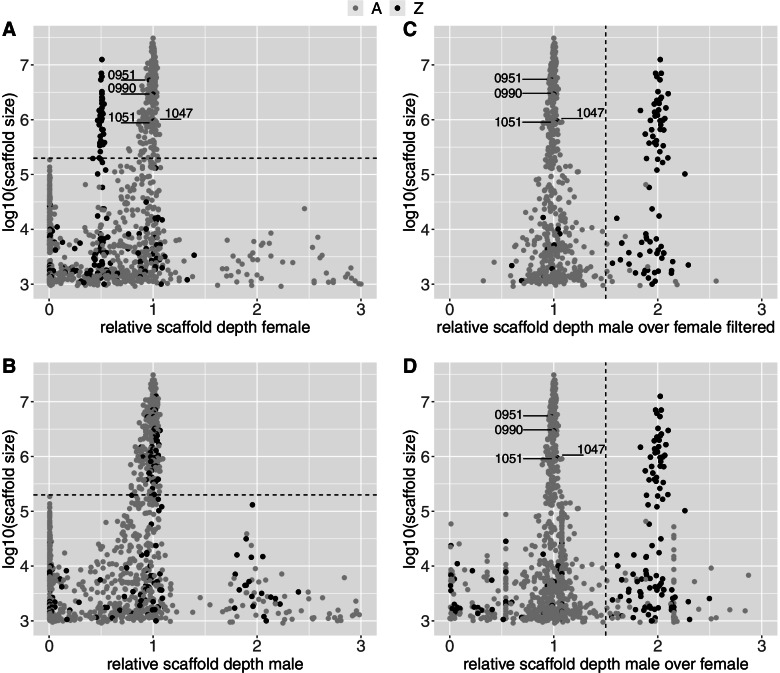
Relative sequencing depth of scaffolds in a female and a male white-tailed eagle. Relative scaffold depth was estimated as mode of scaffold depth / overall genomic depth, which was 195 for the female and 181 for the male. The shading of the dots, representing scaffolds, refer to whether they map to the Z or autosomal (**A**) chromosomes in the golden eagle genome with known chromosomes. **A** Relative depth in the female. **B** The male to female ratio (r_mf_) of relative scaffold depth after filtering (removing scaffolds with relative depth outside the range of 0.25–1.5 in either the male or female). **C** Relative depth in the male. **D** The male to female ratio (r_mf_) of relative depth for all scaffolds. In A and C the dashed line represents the scaffold size threshold value of 198,789 bases (log10 5.29). In A and C, points lower than the threshold value of 198,789 bases displayed high variation for relative depth (Fig. S2). Scaffolds below the threshold in A and B make up 1.1% of data, only 0.0071% is below the threshold and above a relative depth of 3. Dashed line in B and D is 1.5, which is right between expectation for autosomal (1) and Z chromosomes scaffolds (2). “0951”, “0990”, “1047”, and “1051”, in A, B, and D, refers to the scaffolds NW_011950951.1, NW_011950990.1, NW_011951047.1 and NW_011951051.1

The overall modes of depth, 195x for the female and 181x for the male, were used to estimate the relative sequence depth for each position on each scaffold. A clear bimodal distribution of the depths was observed after discarding the shortest scaffolds (< 198,789 bases, log10 < 5.29) (Fig. 1, Fig. S2, Fig. S3) and a good distinction of the expected values for the Z-chromosome (0.5) and the autosomes (1) for the female was observed (Fig. 1A and S2). As also expected, this was not observed for the male, but a few Z scaffolds had a ratio of 2 suggesting occurrence of paralogous regions (Fig. 1C). After the removal of the short scaffolds, 257 scaffolds out of the 1141 scaffolds remained, but covering 98.9% of the full genome in the chromosome-assembled golden eagle genome, which was used as baseline. In the female, 36 scaffolds comprising ~ 75.2 Mb had a relative depth close to 0.5 (from 0.466 to 0.533), all from the golden eagle Z-chromosome. In comparison, 211 scaffolds (1.0947 Gb) had a relative depth around 1 (from 0.764 to 1.062), whereof 207 were autosomal. The remaining four scaffolds (NW_011950951.1, NW_011950990.1, NW_011951047.1 and NW_011951051.1) mapped to the Z chromosome, comprising ~ 10 Mb or 0.91% of the scaffolds identified as autosomes (see Table 2 and Table S1 for all numbers).

The expected male to female ratio (*r*_*mf*_) of sequence depth is 1 for autosomal and 2 for Z scaffolds. Implementation of *r*_*mf*_ for the scaffolds revealed an even clearer split between the Z and the autosomes (Fig. 1B), particularly after removing the primarily small scaffolds with relative depth outside the credible range of 0.25–1.5 in either the male or female. This left 618 scaffolds that accounted for 99.53% of the total sequence (Fig. 1D). Thereof 93 had *r*_*mf*_ > 1.5, consistent with the expected depth of Z scaffolds. Of these, 79 (76.2 Mb) identified as Z and 14 (0.09 Mb) as autosomal chromosomes in the golden eagle genome. We observed 525 scaffolds with *r*_*mf*_ < = 1.5, consistent with the expected depth of autosomes. Of these, 512 scaffolds (1100.7 Mb) identified as autosomes and 13 (10.05 Mb) as Z in the golden eagle genome (four of these 13 were also scaffolds NW_011950951.1, NW_011950990.1, NW_011951047.1 and NW_011951051.1).

### Heterozygosity

**Fig. 2:**
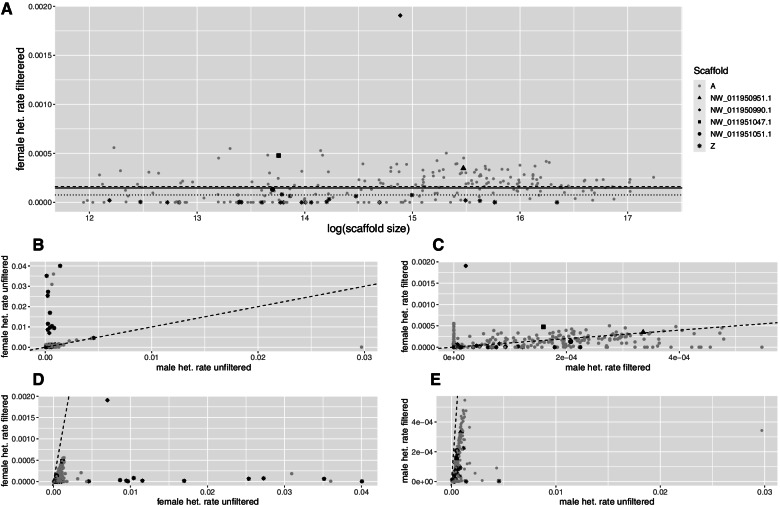
Heterozygosity rate of scaffolds mapped to autosomes and the Z-chromosome in white tailed eagle. Heterozygosity rate as number of heterozygous positions divided by length. **A** filtered heterozygosity rate of the female. The dashed and dotted lines represent the mean filtered heterozygosity rate for autosomal and Z scaffolds, respectively. The full line is for all scaffolds. **B** unfiltered heterozygosity rate for the female plotted against the male. **C** filtered heterozygosity rate for the female plotted against the male. **D** filtered versus unfiltered heterozygosity rate in the female. **E** filtered versus unfiltered heterozygosity rate in the male. In all plots shape and color reflect scaffold type; grey dot is autosomal; black star Z-chromosomal; triangle, diamond, square and black dot are scaffolds NW_011950951.1, NW_011950990.1, NW_011951047.1, NW_011951051.1, respectively, which all show high heterozygosity in the female and have a relative depth as being autosomal. In **B** through **E**, dashed lines represent the identity line (slope = 1)

After filtering, where low quality and spurious sites based on deviation from statistical expectation were removed, only 32% of scaffolds (365 of 1141), covering 97.5% of the genome, had at least one heterozygous genotype in either of the two individuals. Slightly fewer heterozygous sites were observed in the female (288) than in the male (300). The majority of the scaffolds with no heterozygous sites mapped to the Z (80% in the female, corresponding to 30% of the Z chromosome; 77% in the male, covering 23% of Z). The Z had generally fewer heterozygous sites after filtering (Table 1, Supplement Figs. 3 and 4), but a majority of the autosomal scaffolds lack heterozygous sites (67, 1.1% in size). Furthermore, there were more autosomal scaffolds than Z’s. Seventy-seven scaffolds (52.5 Mb, ranging from 1.5–5565 kb) had no heterozygous genotypes in the female but a minimum of one heterozygous genotype in the male and ten of those scaffolds (10.1 Mb) mapped to the Z-chromosome in the golden eagle genome. Aside the larger fraction of the Z scaffolds which had no variation on Z, about 62% of the Z-chromosome in the female had also considerably fewer heterozygous sites than the male (supplement Fig. 3), but some show autosomal levels of heterozygosity in the female (separately marked in Fig. 2A). Four of these scaffolds also exhibited autosomal levels of depth in the female (Fig. 1) and two of those scaffolds (“NW_011950951.1” “NW_011950990.1”) in the female had the highest number of heterozygous sites (1823, 5568), followed by NW_011951047.1 which had 450 sites.

**Table 1:** Information about heterozygosity for a female and male. Heterozygosity for each of the male and female for scaffolds that map to the A and Z in the golden eagle genome with known chromosomes. Numbers of heterozygous (hets.) sites, scaffolds and windows of size 50,000 bases. Total number of scaffolds and 50 k windows were 1141 and 23,585 respectively

	Female Z	Female A	Male Z	Male A
Proportion of heterozygous sites before filtering	0.00534	0.00067	0.00050	0.00065
Proportion of heterozygous sites after filtering	0.00010	0.00018	0.00007	0.00019
Scaffolds with no heterozygous sites	134 (80%)	720 (74%)	130 (77%)	712 (73%)
Size of scaffolds with no heterozygous sites (kb)	26,625 (31%)	55,018 (5%)	20,010 (23%)	65,907 (6%)
Scaffolds with heterozygous sites	34	254	38	262
Heterozygous sites per window (50 kb) (median)	0	1	0	2
Standard deviation per window (50 kb)	43.0	12.3	8.1	12.2
Coefficient of dispersion (CD)	360.6	16.1	20.2	15.6
Windows with no heterozygous site	1398	10,264	1267	9857
Windows with heterozygous sites	304	11,619	435	12,026

The four Z chromosomal scaffolds that had a male-like pattern of autosomal depth and heterozygosity in the female were further analysed in windows of 50Kb, as heterozygous sites can be restricted to small parts of the scaffold (Fig. S6). An examination of the number of filtered heterozygous sites per 50Kb window in these four scaffolds in the female, showed that NW_011950951.1, NW_011950990.1 consisted of either 1 or 2 continuous regions, whereas the other two were more fragmented.

The average heterozygosity per scaffold, prior to filtering, was > 10-fold higher in the female than the male for the Z-chromosome (Table 1), and several scaffolds were even higher (Fig. 2B). The filtering removed most of this excess heterozygosity in the female (Fig. 2C, D and E). As the pattern of excess heterozygosity in the female was primarily seen in Z rather than autosomal scaffolds, we postulate that these instances might represent the mapping of diverged homologous reads from the W chromosome.

Overall, the distributions of heterozygous sites per window was similar for the male and the female and almost half of the windows had no heterozygosity (49% in the female and 47% in the male). When the windows were grouped by Z and autosomes, a difference between the sexes was observed for the Z-chromosome (Table 1 and Fig. S4 and S5). As expected, there was a higher proportion of windows on Z with no heterozygous sites in the female (82%) than in the male (74%) (*P* = 6.111*10^− 8^, Fishers exact test). However, the 10 most variable 50 kb windows in the female, with rate of heterozygous sites ranging from 0.17–1.73% all came from the scaffold NW_011950990.1 which map to Z. The window in the male with the largest rate of heterozygous sites had 0.15%. This difference in the distribution of heterozygosity per 50 kb windows on the Z chromosome per sex is also reflected in the average number and standard deviation of heterozygous genotypes per window, which was larger in the female Z (5.1 and 43) than in the male Z (3.2 and 8.1), whereas no differences were observed in these descriptive statistics for the autosomes. This means that the distribution of heterozygous genotypes was more clumped for Z in the female (Coefficient of dispersion, CD = 360.5) than in the male (20.2) and the autosomes of both sexes (~ 16) (Table 1).

### Mapping

Mapping the 1141 scaffolds from the golden eagle scaffold assembly to the chicken genome, using LASTZ, resulted in 110 scaffolds (86.5 Mb) correctly assigned to the Z-chromosome, and 940 scaffolds correctly assigned to autosomes, according to the comparison of mapping to the golden eagle chromosome-assembled genome. On the other hand, 33 scaffolds (0.59 Mb, amounting to 0.69% of the total length of scaffolds) were wrongly assigned to the Z-chromosome, and 58 scaffolds (0.27 Mb, 0.024%) were wrongly assigned to autosomes (Table 2).

**Table 2:** Classification of scaffolds identified as Z or autosomal scaffolds. Classification for each of the approaches: depth, heterozygosity, LASTZ, and SNP-loading analysis. The identification was found by comparison to the golden eagle genome bAquChr1.2 (GCA_900496995.2) with known chromosomes. Results for the different methods are given in a) for total size of scaffolds (bp), and in b) for the number of scaffolds, missing is obtained by comparison with the golden eagle scaffold assembly

		Depth		Heterozygosity		LASTZ		SNP-loading	
a)		Z	A	Z^a^	A	Z	A	Z	A
	Z	76,239,124	10,056,095	–	60,214,856	86,569,008	270,522	69,355,267	13,642,226
	A	93,786	*1100,765,118*	–	1,050,885,219	597,603	1,105,305,943	11,720,756	1,078,283,284
	*Total*		*1,187,154,123*		*1,159,757,217*		*1192,725,744*		*1,173,001,533*
	*Missing*		*5,571,621*		*29,104,198*		*0*		*19,714,211*
b)	Z	79	13	–	34	110	58	28	12
	A	14	512	–	254	33	941	9	231
	*Total*		618		*365*		*1141*		*280*
	*# NA*		*523*		*776*		*0*		*861*

^a^values not assigned due to lack of heterozygosity on the Z chromosome

### PCA

**Fig. 3:**
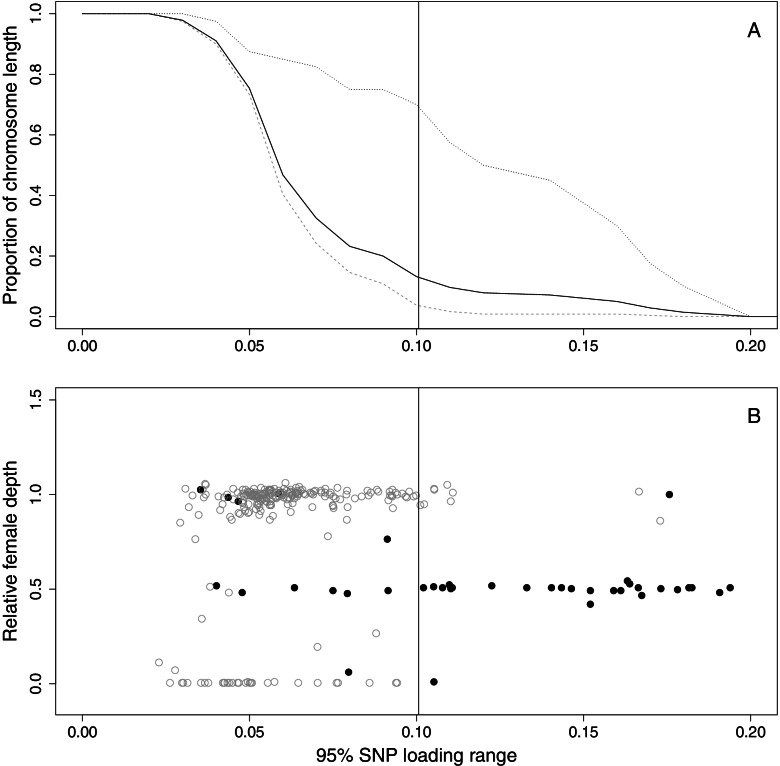
Discrimination of the sex-chromosomes with respect to SNP-loading and genomic information. **A** Proportion of the genome plotted against 95% range of SNP-loading values for PC1. Dotted black line presents the proportions for the Z-chromosomal scaffolds, dashed grey line the values from the autosomal and full black line the values for all scaffolds pooled. **B** Relative scaffold depth for the female plotted against the 95% range in SNP-loadings. Open grey circles refer to autosomal scaffolds and full black dots to Z-chromosomal scaffolds. The vertical line, in both plots, represents 3 SDs above the mean. For legibility, the upper value on the y axis was set to 1.5 in panel B. Two scaffolds, one autosomal and one Z-chromosomal, had relative depth greater than 1.5 (both > 15), with a SNP loading range around 0,025 and 0,15, respectively

The analysis of the loadings of 164,952 SNPs from the PCA analysis (Fig. S1), based on 133 RADseq individuals with an average sequencing depth per site of 2.25 per individual, was limited to the 280 scaffolds (40 Z and 240 autosomal) that had more than 50 SNPs (accounting for 98.3% of the genome). We calculated the 95% range of SNP-loadings on PC1 (i.e., the quantiles 0.025 and 0.975) in our attempt to identify scaffolds belonging to the Z, using a threshold (0.1006) that corresponds to 3 standard deviations above the mean (Fig. 3A and B, Table 2). Of the scaffolds included in this analysis, 28 (78%) scaffolds from the Z-chromosome were above this threshold, accounting for 69.3 Mb (83.6% of the total length of Z scaffolds used in this analysis). In contrast, only 9 (3.75%) of the autosomal scaffolds were above the threshold, amounting to 11.7 Mb (1.1% of the total length of autosomal scaffolds used in this analysis). Thus, the range of PC1 loadings provides some discriminatory power to distinguish Z from autosomal scaffolds.

### Comparison of the four methods

**Fig. 4:**
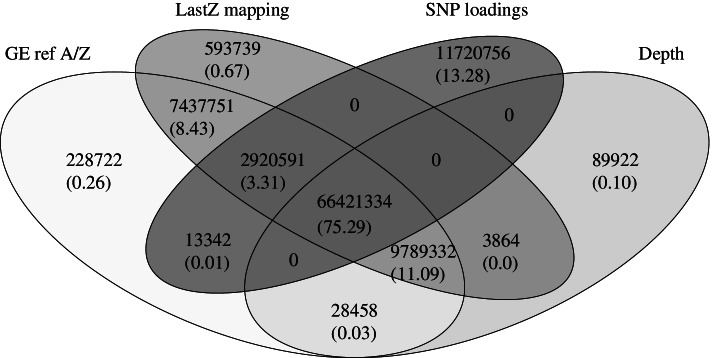
Venn diagram summarizing the size of scaffolds in bases identified as Z-chromosome with the three different analyses: mapping, depth and SNP-loadings. The Z-chromosomal scaffolds were assigned by mapping the genome with scaffolds to the genome with known chromosomes. Values in parentheses represent percentage size compared to the size of the known Z-chromosome. Notice that the percentage found by mapping the golden eagle scaffold assembly to the golden eagle genome is only 98.42%

Using chromosome assignments obtained by mapping the golden eagle scaffold assembly to the golden eagle genome with assigned chromosomes, the most successful method was mapping to the chicken genome, finding 98.12% of the expected size (Table 2, Fig. 4). In second place was the depth analysis with 86.41% and, in third, the SNP-loading with 78.61%. Heterozygosity was poorly suited to find Z-chromosomal scaffolds as a large fraction of scaffolds had no variation, and some Z-chromosomal scaffolds were found to be highly variable in the female (likely due to the mapping of reads that belong to the W chromosome). Depth, mapping to the chicken and SNP-loading all found false positives, i.e., autosomal scaffolds that were categorised as Z-chromosomal scaffolds (0.09, 0.59 and 11.72 Mb, respectively). All approaches resulted in false negatives i.e., Z-chromosomal scaffolds categorised as autosomal (Table 2), but least with mapping to the chicken (0.27 Mb), whereas depth, heterozygosity, and SNP-loading had 10.05, 60.21 and 13.64 Mb of false negatives, respectively. Forty-five very short Z-chromosomal scaffolds (with a total length of 0.22 Mb) were not found by any analysis, and were only found when the golden eagle scaffold assembly was mapped to the golden eagle with known chromosomes. Mapping of the golden eagle scaffold assembly to the golden eagle with assembled chromosomes revealed 98.42% of the whole known Z-chromosome (Table 2, Fig. 4). Though the goal of the study was to evaluate the approaches separately, a combined analysis (Fig. 4) where at least two of three approaches (e.g., depth, mapping to the chicken, and SNP-loading) were compared, detected between 75.29–86.29% of the size of the Z-chromosome of the golden eagle genome, and only the approach combining depth and mapping to the chicken found false positives, which was less than < 0.01% of the size of the golden eagle Z-chromosome.

## Discussion

Three of the four methods evaluated in this study; the relative depth, mapping to chicken, and SNP-loadings, were able to detect a high fraction of the Z-chromosome of the white-tailed eagle that had been mapped on the golden eagle scaffold assembly. The success of the methods varied as they may be affected differently by the small population size of the study species. The approaches applying heterozygosity and PCA are expected to be more affected by a small population because they analyse genomic and population diversity, whereas depth and mapping are expected to be less affected by the low diversity in a small population.

The mapping of contigs to genome sequences from a distantly related species such as golden eagle to chicken can be problematic due to architectural changes such as translocations and inversions. Minor mismatches, e.g., transposable elements and mutations, may further impact the success of finding the Z-chromosome. However, sex chromosomes may be well preserved in birds e.g., Xu and Zhou [19], and this effect seems to be minimal in the case of mapping the golden eagle scaffold assembly to the chicken with a split time > 80 million years [5].

The Z scaffolds that were not detected using the SNP-loading approach are likely due to parts of the Z-chromosome that lack variation, or that share homologous regions in the distinct sex chromosomes and do not contribute to the difference between the sexes in the PCA-plot. The PCA approach found few false positives, possibly due to the lack of a precise distinction between the range of loadings observed for the autosomal and Z-chromosomal scaffolds. Considering the information from the mapping it is clear that the Z-scaffolds have higher impact, as most false positives were just above the threshold of three SDs (i.e., 0.10 95% SNP loading range), and only two autosomal scaffolds were larger than ~ 0.11 comprising only a total size of 1.73 Mb, or 14% of the false positives. The SNP-loading approach also found false negatives (Table 2) and we feel this deserves further research.

Here, the approach of looking at all scaffolds in a single PCA was used, but this could potentially be optimized by using sliding windows [41] to identify signals different from the overall population signal. However, this also requires diversity on the homogametic sex chromosome in males compared to females, which may be lacking in small populations such as in the Icelandic white-tailed eagle.

Inspection of the heterozygosity for all scaffolds revealed that it is difficult to distinguish between autosomal and Z-chromosomal scaffolds without any prior knowledge. However, there was a difference in the average heterozygosity between autosomal and Z-chromosomal scaffolds, especially in the female. Small populations, such as the white-tailed eagles in Iceland [9], have reduced heterozygosity and long runs of homozygosity were observed on the Z-chromosome and the autosomes, making it more difficult to distinguish among the chromosomal types. Furthermore, there is a clear overlap in scaffolds with some heterozygosity which might belong to PAR and non-homologus regions, e.g., due to inversions, on the Z- and W-chromosomes. PAR and the nonrecombining homologous regions, could explain some deviations in the prediction of the Z-chromosome in the SNP-loading analysis but these regions are probably small, and thus won’t display the signal of an autosome in the depth analysis. Although genome wide information from a single individual can provide assessment of variation within populations, it can be biased due to missing chromosomal fragments and thus the overall success of the method. However, the two high depth individuals here show no clear indication of such deviation, as we obtain most of the Z-chromosome in the analysis.

The relative depth analysis revealed 86.41% of the expected size of the Z-chromosome and found few false positives. Four scaffolds were noted as false negatives in one of the two depth analysis. These four scaffolds (NW_011950951.1, NW_011950990.1, NW_011951047.1, and NW_011951051.1) make up about 10 Mb and show the highest heterozygosity of all Z-chromosomal scaffolds after filtering; their levels are comparable or even higher than observed for the autosomal scaffolds. Three of the four scaffolds showed low 95% SNP-loading ranges (all around 0.05), unlike the scaffolds contributing to the separation of the sexes. One scaffold (NW_011950990.1) had a very high 95% SNP-loading range and very high heterozygosity. This signal in these four Z scaffolds, and position at the end of the Z-chromosome supports that they belong to the pseudo-autosomal regions (PAR) as seen in other birds [29, 42]. In birds, PAR vary greatly in size from just a few Mb to more than 60 Mb [29]. Alternatively, they could represent non-recombining homologous regions (gametologs) [19, 43] which can be expected to have even higher heterozygosity in females than within the recombining Z-chromosomes in the homogametic males or the autosomes, because such regions could have evolved independently for millions of years. Two of the four scaffolds mentioned above, NW_011950990.1 and NW_011951051.1, display a higher heterozygosity ratio in the female compared to the male (17, and 2.5 times higher, respectively), as expected for gametologous regions, whereas the other two NW_011950951.1, and NW_011951047.1, may present PARs, as they display a ratio close to one between the sexes (1.08 and 0.78, respectively). A fully annotated genome of the white-tailed eagle would provide further information about these gametologous regions within the Z- and W-chromosome.

Although depth analysis has shown to be a promising method to identify sex chromosomes [23, 44], it is not error free. Scaffolds belonging to the Z-chromosome can have a depth as high as autosomes, as variance in depth can be large in small scaffolds which may be poorly sampled due to low variation, or the scaffolds include regions from both Z- and W-chromosomes i.e., gametologs and the PAR regions. Here the best approach for identifying the homogametic sex chromosome was mapping to a reference with annotated homogametic sex chromosome. To identify the Z-chromosome, a combination of the mapping with at least one other analysis is recommended as it may result in fewer potential false positives and negatives. Further, it should be noted that the methods used here maybe more applicable in taxa with relative stable sex chromosomes, such as mammals and birds [19, 20], but less effective in taxa such as fish where the sex chromosomes can be less differentiated [21, 22].

The dynamic nature of the Z-chromosome (e.g., songbirds [36, 45]) and potential deviations in synteny may introduce errors into assemblies of two species, however, there is significant and relevant justification for doing so. The approach using a different reference from the study species has successfully been employed in other studies [2–4]. Mapping of our novel white-tailed sequences against the golden eagle assemblies, one assembled to scaffolds, and second assembled to chromosomes, made it possible to evaluate the precision of these approaches to a greater extent. This study highlights potential problems when trying to identify the homogametic sex chromosome that are specific to small populations, which bears importance for the conservation of species at risk.

Even though all known eukaryote species may soon be sequenced [46], it will still be a long time before all parts of their chromosomes have been identified. Thus, it is important to further explore these different methods and how they depend on sequence variation and scaffold sizes, as variation in the different chromosomes will differ due to different effective population sizes and evolutionary histories.

## Conclusion

The best performing approach for identifying the homogametic sex chromosome in the small population of white-tailed eagle was obtained by aligning of the reference genome to a species with annotated sex chromosomes. The second-best approach was an analysis of read depth per scaffold, and third was an analysis of SNP-loadings in a PCA. Identification using genomic diversity approaches. The utility of the SNP-loadings and heterozygotic differences between the sexes suffers likely by the small population size and a recent population bottleneck in the study populations. Evaluation of these methods are highly relevant as genomic regions vary in effective population size and can have different evolutionary histories.

## Methods

### Sample collection, laboratory work and sequencing

Blood samples were collected from white-tailed eagle chicks as a part of an ongoing monitoring program in Iceland since 2001 by the Natural History Institute of Iceland. The sex of the chicks was determined in the field based on tarsus thickness and weight [47]. Three to ten mL of blood was extracted from each chick. The blood was stored in EDTA buffer at − 20 degrees Celsius until DNA extraction.

DNA from blood samples of 135 chicks was extracted using the ThermoFisher GeneJET Whole Blood Genomics DNA Purification Mini Kit following the standard protocol [48]. DNA concentration was estimated using the NanoDrop 1000 and run on 0.7% agarose gels to evaluate the fragment size. Samples with concentration higher than 60 ng/μl were selected for library preparation and sequencing. The 133 of 135 extracts were double digest restriction-site associated DNA sequenced (RADseq) on the Illumina HiSeq2500 (see supplementary text 1 for full description).

A male and female white-tailed eagle were selected for high-depth whole genome shotgun sequencing with two lanes each on an Illumina HiSeqX. Library preparation and sequencing was done at deCODE genetics, using the TruSeq Nano sample preparation method [49].

Two reference assemblies from male golden eagles (ZZ), one in 1142 scaffolds and one assembled to chromosome level (GenBank Assembly Accession numbers: GCA_000766835.1 and GCA_900496995.2, respectively), and a female chicken assembly (ZW) (GenBank Assembly Accession: GCA_000002315.3) were downloaded from NCBI and used in the analysis [12, 50].

### Sequence cleaning and mapping

The white-tailed eagle RADseq data was demultiplexed, sorting sequence reads into individual files, both for forward and reverse sequences using the command ‘process_radtags’ in Stacks version 1.47 [51, 52]. Default settings were used for the RADseq data, applying the option “r” to rescue barcodes and RAD-tags.

After demultiplexing, FastQC [53] was run for quality control. For the RADseq data, an excess of specific sequences (kmers) were removed using AdapterRemoval v2 (version 2.2.2) [54]. The high depth shotgun sequenced individuals were tested in the same way but found no excess of kmers.

The Burrows-Wheeler Aligner (BWA) mem and SAMtools version 0.7.17-r1188 and 1.7, respectively [55, 56] were used to process RADseq and high depth shotgun data and map reads to the golden eagle scaffold assembly of 1142 scaffolds with no identified chromosomes (GCA_000766835.1) [12] using default settings in both instances.

### Four different approaches to find the Z-chromosome - depth, Heterozygosity, mapping and SNP-loadings

**Fig. 5:**
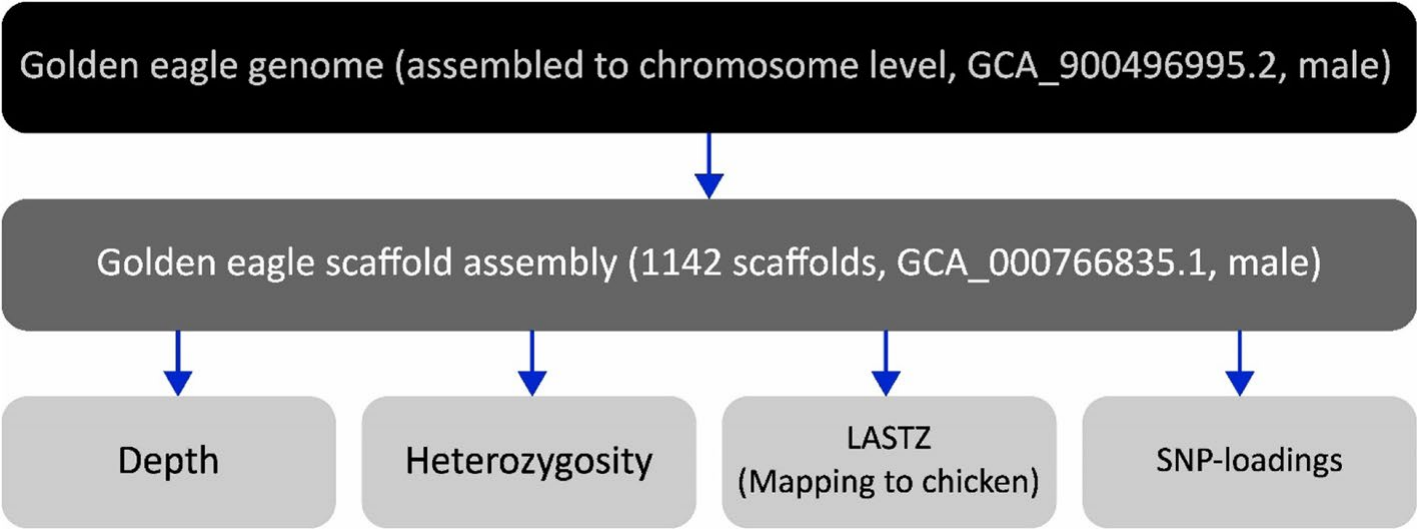
Schematic overview of the methods used to identify the Z-chromosome in a scaffold assembled genome. The golden eagle genome referred to in the dark grey box represents the reference in which we are attempting to identify scaffolds belonging to the Z-chromosome. The golden eagle genome in the black bar is the genome with known chromosomes, used to identify which scaffolds in the dark grey boxed genome probably belong to Z-chromosome (and autosomes) – to use as a reference. The light grey boxes are the four approaches we tested to find the scaffolds belonging to the Z-chromosome: 1) Depth: analysis of difference in sequencing depth between scaffolds in a high depth whole genome sequenced white-tailed eagle female. 2) Heterozygosity: analysis of the difference in heterozygosity per scaffold a high depth whole genome sequenced white-tailed eagle male and female. 3) LASTZ: mapping of the golden eagle reference genome to the chicken genome using LASTZ. 4) SNP-loadings: analysis of SNP-loadings for principal components splitting the sexes, in 133 RADseq white-tailed eagle individuals

Four different approaches were used to identify scaffolds in the white-tailed eagle genome belonging to the Z-chromosome, by comparison with the golden eagle scaffold assembly with no chromosomes (GCA_000766835.1). An assembly consisting of 1141 assembled scaffolds, excluding mtDNA, and a total of 1192,725,744 bp, ranging in size from 913 to 30,727,332 bp with a median of 5587 bp, and average length of 1,045,334 bp (SD 3,203,066 bp). An overview of the methods is presented in Fig. 5 and the data used in each analysis is available in supplementary Table S1.

### Depth

For the high-depth white-tailed eagle sequencing data, the average autosomal sequencing depth was estimated for the male and female separately, as the mode of the number of mapped reads per position across all scaffolds based on results from the command “bedtools coverage” from Bedtools v2.18.2 [57]. Using these averages, 195 for the female and 181 for the male, the relative sequencing depth was calculated for each position in each scaffold for both individuals. The per-scaffold relative sequence depth was then estimated for the female and male separately, as the mode across positions. Positions in autosomal scaffolds are expected to have a relative depth of 1 in both sexes, whereas Z-chromosomal scaffolds are expected to have a relative depth of 0.5 in females and 1 in males. As the estimate of relative depth may be less reliable for smaller scaffolds, the dependency of the relative mode depth due to scaffold size was analysed by calculating the variance in the depths per interval of scaffold sizes, transformed to a log scale. The distribution of the proportions of scaffolds at each interval was summarized with a cumulative percentage curve. In addition, the depth per scaffold was evaluated by comparing the per-scaffold relative sequencing depth between the two individuals: male over female. Scaffolds with a relative sequencing depth below 0.25 and above 1.5 were removed (corresponding to 523 scaffolds, and 0.47% of the genome). This ratio is expected to be around two for Z-chromosomal scaffolds and one for the autosomal scaffolds, as the male has two copies of Z and the female one. Thus, a cut-off was set at 1.5.

### Heterozygosity

Sex differences in heterozygosity were assessed by comparing numbers of heterozygous sites per scaffold based on genotypes of the high-depth white-tailed eagle male and female, called using Graphtyper [58, 59] with default settings. The variation on the Z-chromosome is expected to be ¾ of the autosomes and it should be restricted to the male, except for the PAR and non-recombining homologous regions. As scaffolds vary in length and may include short variable regions, the variation was also analysed per 50 kb window. Genotypes were filtered for quality using vcftools and bcftools version 0.1.15 and 1.7, respectively [60, 61] before counting, using minimum GQ score 20, minimum Q score 1000, missingness 1 (both individuals had to have a valid genotype at the site), mapping quality equal to 60 (MQ), and only biallelic sites. Two additional criteria were applied to remove sites with likely spurious heterozygous genotypes. First, heterozygous genotypes where the number of mapped reads deviated significantly from the mode depth of the scaffold, based on a two-sided Poisson test (*P* < 0.01) were excluded. Second, we used a binomial test to assess whether the proportion of reads in heterozygous genotypes, either in the male or the female, deviated from the 50/50 expectation, using *P* < 0.05 as the exclusion threshold.

### Mapping

In order to assign the short reads from the white-tailed eagle to chromosomes, the 1142 scaffolds from the golden eagle scaffold assembly (which the white-tailed eagle genome had been mapped on) were mapped to the chicken genome, which has assigned chromosomes, using LASTZ [30]. Standard settings were used with the following modifications: ambiguous = iupac, gfextend, chain, gapped. Scaffolds in the golden eagle which mapped better to the Z-chromosome than any other chromosome, measured as most bases mapped, were deemed to belong to the golden eagle Z-chromosome.

### SNP-loadings

A PCA analysis of 133 low-depth RAD sequenced white-tailed eagle individuals was constructed using PCangsd version 1.0 [62], an extension of ANGSD [63], as described below. A clear split between males and females was observed along the first principal component (PC) (Fig. S1). Loadings obtained with PCangsd were used to identify which parts of the scaffolds induced the split, with the “-selection” option [62] and with sites passing the following filters: a minimum 25% of individuals had to have valid genotypes, only unique mapping sites, base quality minimum 20, mapping quality minimum 30, SNP *p*-value 1e-6. ANGSD uses genotype likelihoods to tackle the restrictions of low depth [63, 64]. To assess which scaffolds contributed to the split on the first axis (PC1), a 95% range of loading values for all SNPs per scaffold was calculated using R [65] and compared between scaffolds with more than 50 SNPs. The distributions of the range of loading values were summarized with accumulation curves, combined for all scaffolds, and separately based on the results obtained by the mapping on the autosomes and Z chromosome. Scaffolds were assigned to the Z-chromosome or autosomes depending on whether the range-values were above or below a threshold of three standard deviations from the mean (covering ~ 99% of a normally distributed variable).

### Comparison of the four methods

To evaluate how well the four approaches performed, the golden eagle scaffold assembly (GCA_000766835.1) was mapped to a golden eagle genome with known chromosomes (GCA_900496995.2) using LASTZ with the same settings and cut-off as described previously. In the results, the outcome of this mapping was used as the true chromosome identity of the 1141 scaffolds that was used to assess the accuracy of our four different approaches to identify Z chromosome scaffolds (Fig. 5 and Table 2). A total of 168 scaffolds were assigned to the Z-chromosome, with a total length of 86,839,530 bp (mean = 516,902, sd = 1,509,132, and median = 5236), which is slightly smaller than the Z-chromosome in the newly released genome of 88,216,475 bp (GenBank Assembly Accession: GCA_900496995.2). The autosomal loci mapped to 973 scaffolds of a size of 1,105,886,214 bp (mean = 1,136,574, sd = 3,403,676, and median = 5674). The overlap of these four methods was summarized with the R-package VennDiagram [66].

Summary of the data and further statistical analyses, if otherwise not stated was done using R.

## Supplementary Information

**Additional file 1: Supplementary figures S1-S6** and **supplementary text 1** describing DNA sequencing and extraction.

**Additional file 2: Table S1**. Full raw data file, containing all values used in the calculations.

## Data Availability

The raw dataset supporting the conclusions of this article is available in the DRYAD data repository 10.5061/dryad.v9s4mw6vs. Further, the analysed dataset supporting the conclusions of this article is included in the supplementary.
