# Supplementary material for: Evaluation of four methods to identify the homozygotic sex chromosome in small populations
Source: BMC Genomics. 2022 Feb 24;23:160. doi: 10.1186/s12864-022-08393-z (PMC8867824; doi:10.1186/s12864-022-08393-z)
Supplement: Supplementary file 1 — Additional file 1: Supplementary figures S1-S6 and supplementary text 1 describing DNA sequencing and extraction. [file 12864_2022_8393_MOESM1_ESM.docx]

## Supplementary


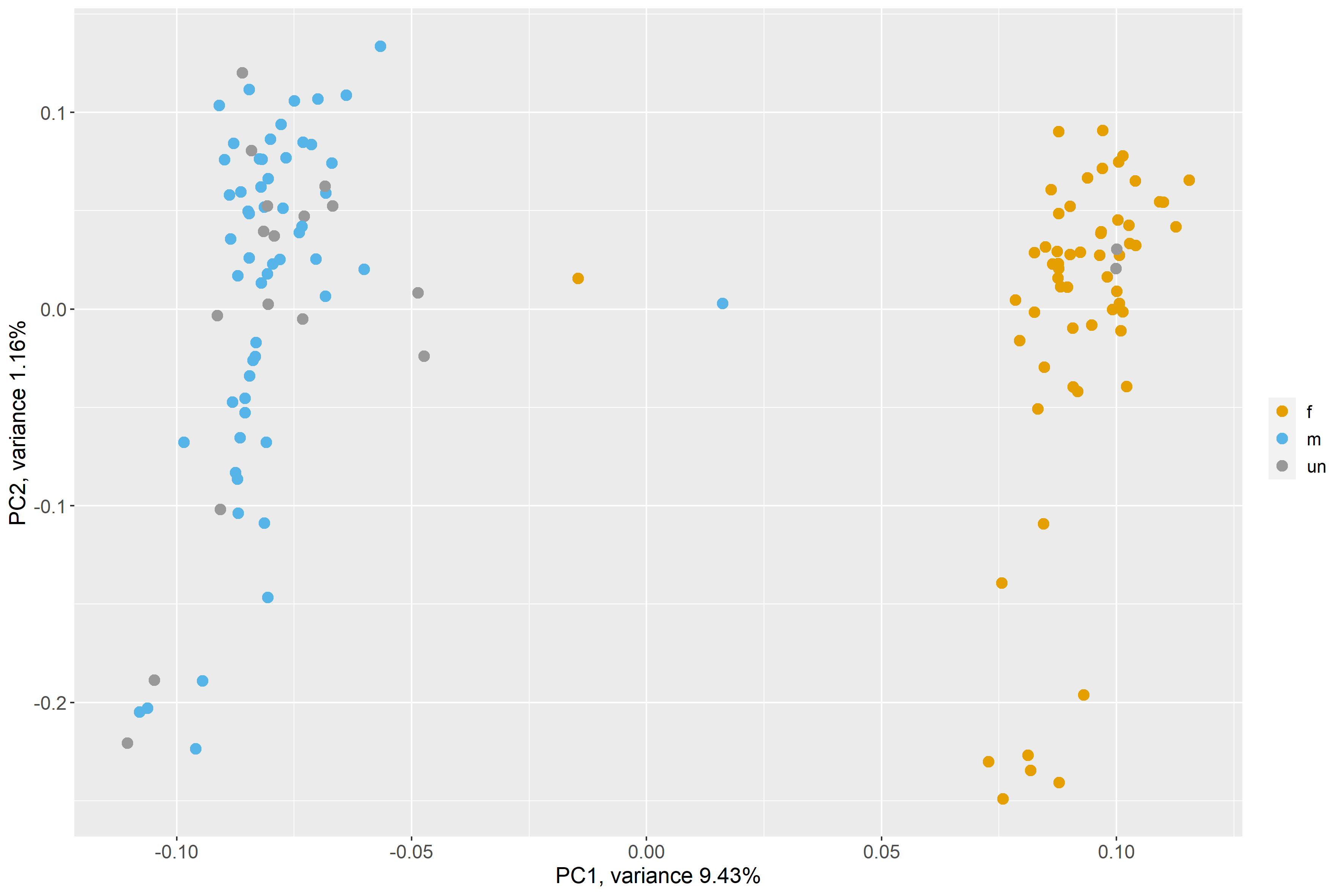


Figure S1. PCA made with PCangsd (Meisner & Albrechtsen, 2018) displaying genetic split between males and females for 133 individuals based on 164,952 SNPs in the Icelandic population. Orange=females (f), blue=males (m), grey=unknown (un), morphologically sexed in the field.


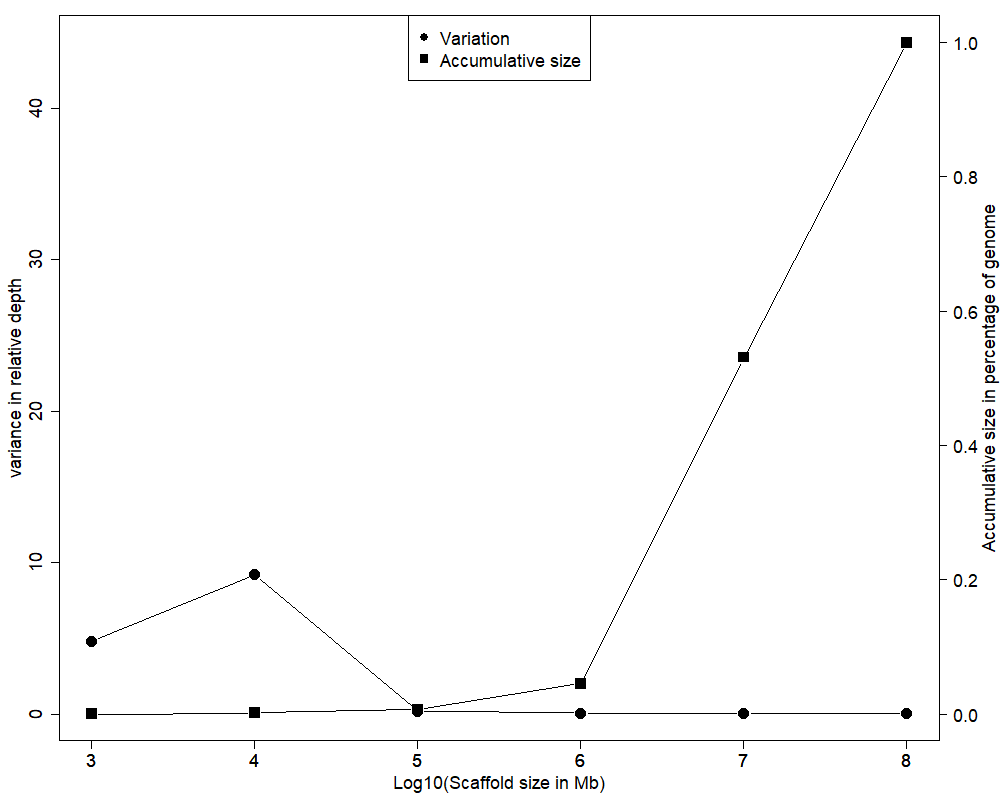


Figure S2. Variation in relative depth in comparison with scaffold size and proportion of the genome. Left y axis with dots show the variance in relative depth between scaffolds per log value, right y axis and square points show the accumulative proportion of the genome.


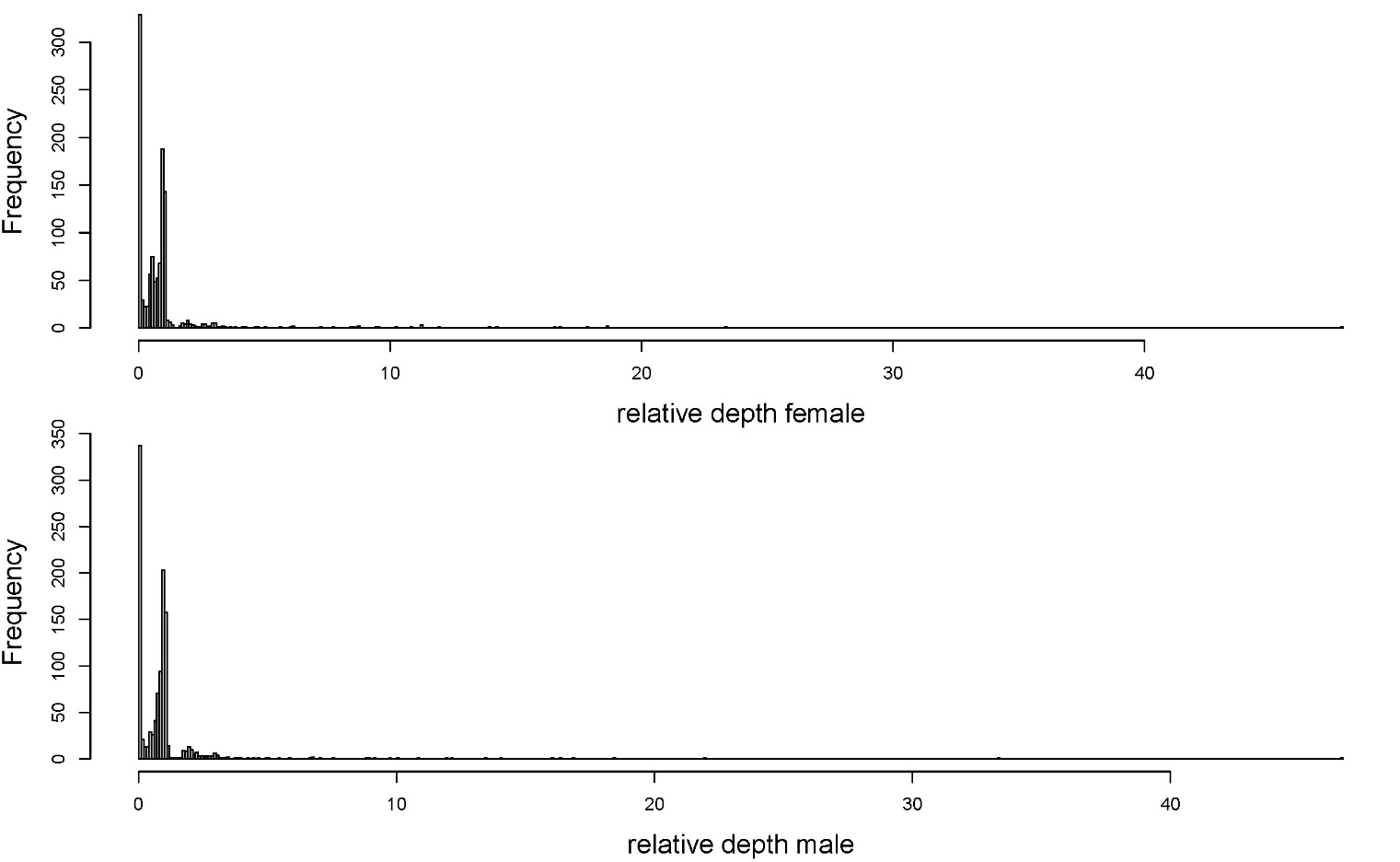


Figure S3. Relative mode depth for the female (top) and male (bottom) for all scaffolds.


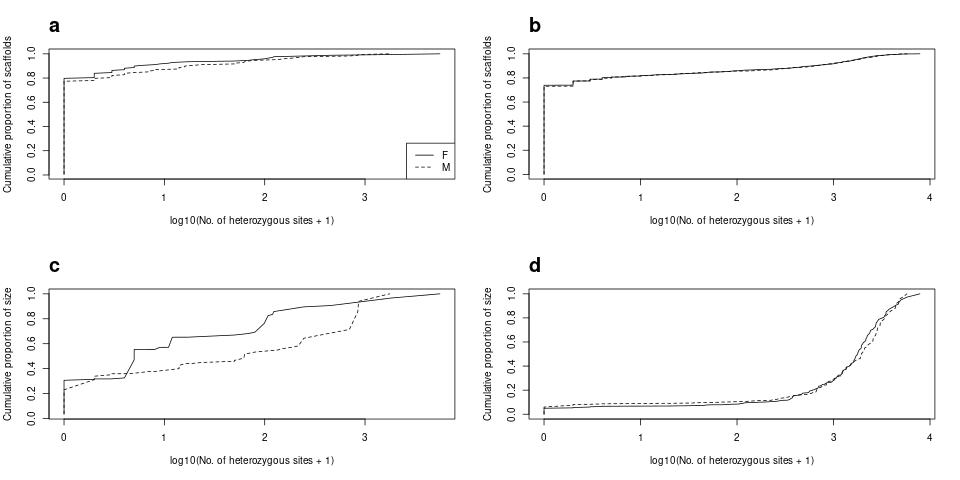


Figure S4. Cumulative proportion of heterozygotic sites and of their chromosomes per scaffold, in the genome of the high depth white-tailed eagle female and male. Proportion of scaffolds on the Z-chromosome (a) and autosomes (b) and proportion of total size of the Z chromosome (c) and autosomes (d). F: female, M: male.


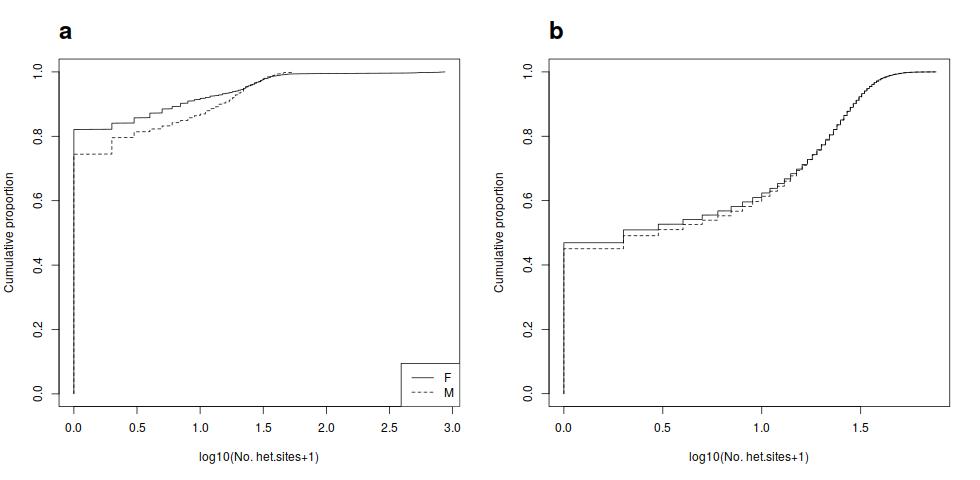


Figure S5. Cumulative proportion of heterozygotic sites in windows of a size of 50 kb in the genome of the two

white-tailed eagles. a) Z-chromosome, b) autosomal chromosomes. F: female, M: male.


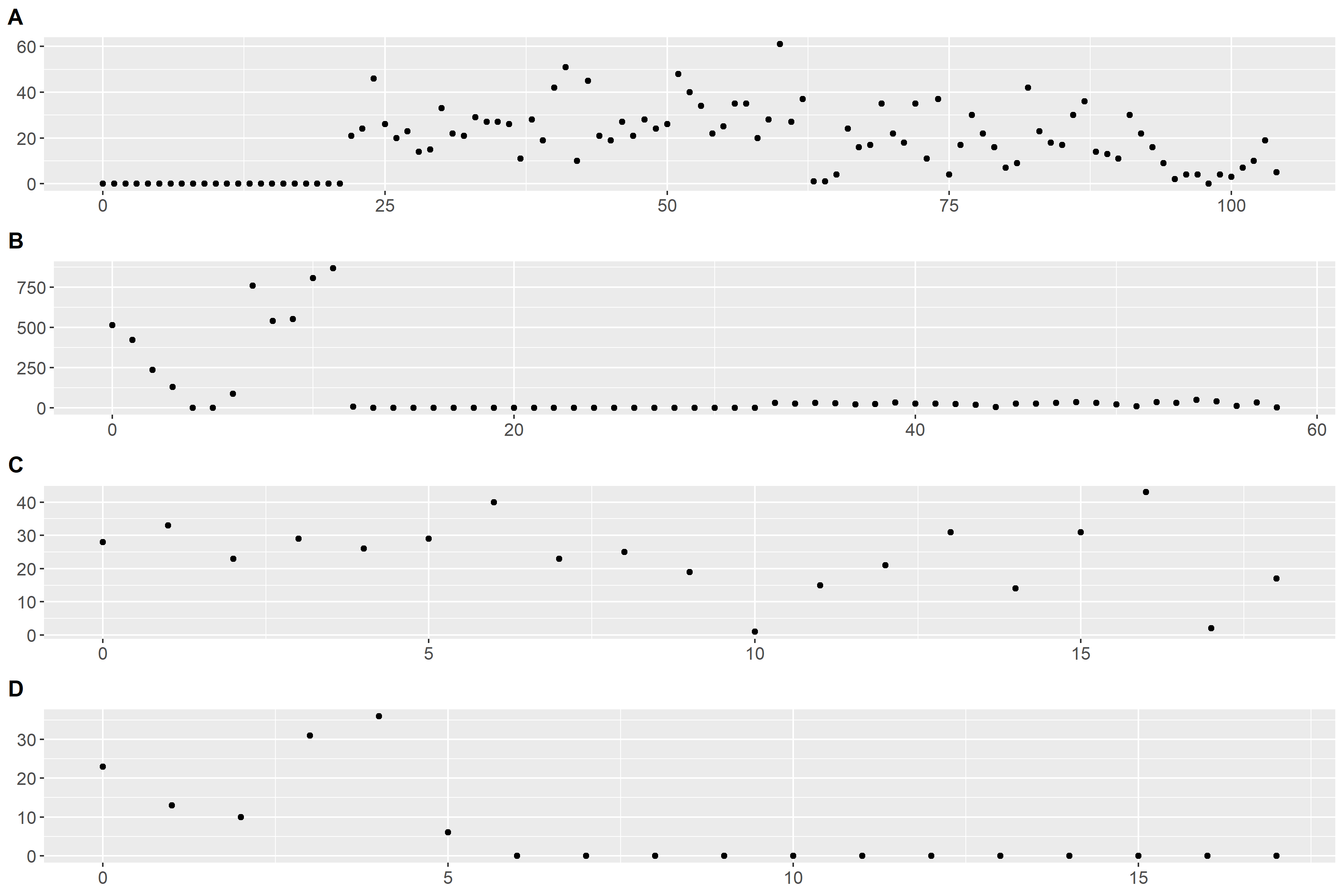


Figure S6. Heterozygosity per window (50 Kb windows) along the scaffold for A) NW_011950951.1, B) NW_011950990.1, C) NW_011951047.1, D) NW_011951051.1.

### Supplementary Table 1

See separate file.

Table S1. Full raw data file, containing all values used to refer results in the paper. “golden_contig”: Scaffold name in golden eagle scaffold assembled genome; “chicken_contig_best”: Chromsome name in chicken genome that the "golden_contig" mapped best to; “mapped_bases_best”: Number of bases of "golden_contig" that mapped to the reported chicken chromosome; “mapped_reads_best”: Number of continues reads of "golden_contig" that mapped to the reported chicken chromosome; “chicken_contig_secondbest”: Chromsome name in chicken genome that the "golden_contig" mapped second best to; “mode_M_WZ”: Mode of sequencing depth of high depth male of "golden_contig"; “mode_F_XA”: Mode of sequencing depth of high depth female of "golden_contig"; “sd_M_WZ”: Standard deviation of mode of sequencing depth of high depth male of "golden_contig"; “sd_F_XA”: Standard deviation of mode of sequencing depth of high depth female of "golden_contig"; “length_golden_contig: Length (number of bases) of "golden_contig"; “X5pctile”: SNP loading of the 5. percentil of "golden_contig"; “X95pctile”: SNP loading of the 95. percentil of "golden_contig"; “het_m_all”: Heterozygoes sites with no filtering in the high depth male in the "golden_contig"; “het_f_all”: Heterozygoes sites with no filtering in the high depth female in the "golden_contig"; “het_m_filtered”: Heterozygoes sites with filtering as described in the paper in the high depth male in the "golden_contig"; “het_f_filtered”: Heterozygoes sites with filtering as described in the paper in the high depth female in the "golden_contig"; “TrueChromosome”: Chromsome name in golden eagle chromosome assembled genome that the "golden_contig" mapped to; “TrueChromosomeZorA”: Separation of the chromosomes in "TrueChromosome" into autosomes and Z-chromosome.

### Supplement text 1 - ddRAD library preparation and sequencing

The 133 samples were prepared for double digest restriction-site associated DNA sequencing (ddRADseq) using modified protocols from Elshire et al. [61] and Peterson et al. [62]. Total genomic DNA (100-500 ng) was sequentially digested using the restriction endonucleases Sau3AI (1U) and ApeKI (2U), respectively, each for four hours at manufacturer (NEB) recommended temperatures in NEB Buffer 4. Digested DNA (100 ng) was ligated to adapters (sequences in Elshire et al. [61]) containing unique combinatorial barcodes (16 unique 5 bp barcodes for ApeKI adapters and five unique 6 bp barcodes for Sau3AI adapters) for each individual (barcode and adapter sequences in Supplementary Information S1) using T4 DNA ligase (NEB) in supplied buffer at 21°C for four hours. Ligation reactions contained a 6:1 molar excess of adapter to fragmented DNA, calculated using the mean fragment size determined from an agarose gel. Ligated DNA was pooled and purified using magnetic beads (Macherey-Nagel NGS clean-up and size selection) following the manufacturers protocol. Size selection of ligated DNA fragments was performed on a Pippin Prep (Sage Science) with 2% ethidium-free agarose gels and external size standard. The narrow range setting included a mean fragment size of 350 bp ± 18 bp. The eluate was split among eight PCR reactions and amplified using the primers and PCR conditions as in Elshire et al. [61]. Each PCR reaction had a total volume of 25 μL containing; 1x OneTaq Master Mix with Standard Buffer (NEB), 0.5 mM each primer, and 8 μL template DNA. PCR products were pooled and purified using magnetic beads before quantification using a SYBR Gold fluorometric assay (protocol in Supplementary Information S2). The library was prepared for sequencing following manufacturer’s instructions with a final concentration of 38 nM. The library was sequenced on an Illumina HiSeq2500 using the Illumina TruSeq kit (2x125bp) following the manufacturer’s instructions. The sequencing was done on one lane and obtained 303 million unambiguous PE reads.
